# Altered B cell compartment associated with Tfh cells in children with Henoch-Schonlein Purpura

**DOI:** 10.1186/s12887-021-02873-z

**Published:** 2021-09-13

**Authors:** Ning Zhang, Ge Tian, Yuanyuan Sun, Jing Pan, Wei Xu, Zhe Li

**Affiliations:** Department of Hematology, The First Affiliated Hospital of Jinzhou Medical University, No.2 Renmin Street, Liaoning 121000 Jinzhou, People’s Republic of China

**Keywords:** Henoch-Schonlein purpura, B cells, CXCR5, subsets, Tfh

## Abstract

**Aim:**

IgA-producing B cells have been found to be associated with children diagnosed with Henoch-Schonlein purpura (HSP). The aim of the present study was to determine whether children with HSP possess altered B-cell subsets.

**Methods:**

A total of 14 children diagnosed with HSP and age- and sex-matched healthy controls (HCs) were enrolled in our study. Peripheral blood mononuclear cells were isolated, and the percentage and absolute number of B-cell subsets and Follicular helper T (Tfh) cells were determined by flow cytometry. Finally, Spearman’s correlation coefficient was used to analyse the correlation between the percentage of Tfh cells and B-cell subsets.

**Results:**

We found that compared to HCs, the frequency and absolute number of total B cells were significantly higher in children with HSP, but the percentages of plasma cells and naïve B cells were significantly lower. A significantly increased percentage and absolute number of memory nonswitched B cells were found in children with HSP compared with HCs. We observed that the expression of C-X-C chemokine receptor type 5 (CXCR5) on total CD4^+^ T cells and the percentage of CD4^+^CXCR5^+^ cells were significantly increased in patients with HSP. Moreover, significantly correlations between Tfh cells and various B-cell subsets were observed.

**Conclusions:**

Our study showed a Tfh-cell-associated altered B cell compartment in children with HSP.

## Key notes

1. Altered B-cell populations were observed in children with HSP.
2. Increased Tfh frequency was found in children with HSP.
3. Significant correlations were found between Tfh cells and B-cell subsets.

## Introduction

Henoch-Schonlein purpura (HSP), the most common type of childhood vasculitis, is characterized by the deposition of systemic IgA immune complexes in the walls of small vessels [1]. It is estimated that 1/5000 children acquire HSP per year [2]. Although considered to be a self-limiting condition, HSP is manifested by skin purpura, arthritis, abdominal pain and renal involvement, and the exact pathogenesis of HSP remains unknown.

HSP is a systemic inflammatory disease that is characterized by leucocyturia, haematuria and proteinuria. Inflammatory cytokines, such as interleukin (IL)-6, IL-10 and IL-17, have been reported to be involved in the pathogenesis of HSP[3–5]. In addition, elevated serum IgA and IgA-related immune complexes have been reported to play an essential role in the pathogenesis of HSP. Recently, IgA-producing B cells were also found to be related to children with HSP[6].

Dysregulated B-cell subpopulations have been found in a variety of autoimmune diseases, such as IgG4-related disease and primary Sjӧgren’s syndrome[7]. The production of high-affinity antibodies is believed to result from interactions between B cells and T follicular helper (Tfh) cells, and an increased number of circulating Tfh cells has been correlated with the severity of autoimmune diseases[8, 9].

Whether children with HSP possess altered B cell subsets remains unknown. Therefore, in the present study, we sought to determine differences in the population of B cells and related subsets (including plasma cells, naïve B cells, memory nonswitched B cells and memory switched B cells) between children with HSP and healthy control patients and to correlate these differences with the percentage of circulating Tfh cells. Our results may provide insight into the potential role of B-cell subsets and circulating Tfh cells in the pathogenesis of HSP.

## Materials and methods

### Clinical demographics

A total of 14 children diagnosed with HSP and age- and sex-matched HCs from May 2017 to March 2018 at The First Affiliated Hospital of Jinzhou Medical University (Jinzhou, China) were enrolled in our study. The average age of all the patients was 7.5 ± 2.1 years, and that of the healthy controls was 7.2 ± 1.9 years. The HSP patients and HCs exhibited the following characteristics: a sex ratio of 8/6 and 7/7 (male/female), respectively; an average white blood cell count of 11.5 ± 2.3 and 6.5 ± 1.8, respectively; and an average lymphocyte count of 2.72 ± 0.85 and 3.52 ± 0.89, respectively. The basic characteristics of the HSP patients and HCs are presented in Table 1. All HSP patients were diagnosed according to modified criteria[10]: in brief, the presence of purpura or petechiae with lower limb predominance plus at least one of the following four features: (1) abdominal pain; (2) arthritis or arthralgia; (3) leukocytoclastic vasculitis or proliferative glomerulonephritis with predominant deposition of IgA on histology; and (4) renal involvement. The following exclusion criteria were used: all the included HSP patients did not receive any treatment or suffer from any other diseases within the last 6 months prior to enrolment in the present study. All blood samples were obtained at the time of diagnosis with acute HSP. Written informed consent was obtained from the enrolled children or their legal guardians. The study was approved by the ethics committee of the First Affiliated Hospital of Jinzhou Medical University.

### Isolation of Peripheral Blood Mononuclear Cells (PBMCs)

Fresh blood samples were collected from HSP patients and HCs in ethylenediaminetetraacetic acid (EDTA) tubes. The samples were diluted 1:1 with Hanks’ solution and carefully layered on Ficoll-Hypaque to enable the formation of density gradients. The interphase cell layer was transferred to new tubes after centrifugation at 800 g for 20 min at room temperature (RT). The cells were then washed with Hanks’ solution twice. The viability of the isolated PBMCs was determined by trypan blue exclusion staining, and the total viability of PBMCs was reported as > 95 %. The harvested PBMCs were immediately used in flow cytometry experiments.

### Flow cytometry

The viable cells were counted and resuspended in cell staining buffer at 5 ⋅ 10^5^ cells/tube. All the antibodies used in the present study were purchased from BioLegend, Inc. (San Diego, CA, USA). For B-cell subset staining, CD19-FITC (clone: HIB19)/IgD-PE (clone: IA6–2)/CD38-PerCP-Cy5.5 (clone: S17051A)/CD27-APC (clone: O323)/CD3-APC-Cy7 (clone: UCHT1) were used. For Tfh-subset staining, CXCR5-FITC (clone: J252D4)/CD3-PE (clone: OKT3)/PD-1-PerCP-Cy5.5 (clone: EH12.2H7)/CD4-APC (clone: A161A1) were used. The following antibodies were purchased from BioLegend, Inc. (San Diego, CA, USA): isotype control FITC-conjugated mouse IgG1, κ (Cat# 400,108); PE-conjugated mouse IgG1, κ (Cat# 400,112); PE-conjugated mouse IgG2a, κ (Cat# 400,211); PerCP-Cy5.5-conjugated mouse IgG1, κ (Cat# 400,150); PerCP-Cy5.5-conjugated mouse IgG2a, κ (Cat# 400,251); APC-conjugated rat IgG2b, κ (Cat# 400,611); APC-conjugated mouse IgG1, κ (Cat# 400,122); and APC-Cy7-conjugated mouse IgG1, κ (Cat# 400,128). Anti-CD16/32 antibody was added to the isolated lymphocytes to inhibit the Fc receptor at RT for 10 min. The cells were incubated with the indicated concentrations of antibodies according to the manufacturer’s instructions at RT for 15 min and then washed; 80,000 to 100,000 total events were collected using a BD FACS Aria II flow cytometer (BD Biosciences, Franklin Lakes, NJ, USA). FlowJo Software (FlowJo LLC, Ashland, OR, USA) was used for data analysis. Lymphocyte subset absolute counts were measured by dual platform.

### Statistical analysis

SPSS 21.0 software was used for data analysis. To determine the significance between the two groups, the Student’s *t* test was applied to data conforming to a normal distribution, and the Mann–Whitney test was applied to data not conforming to a normal distribution. Spearman’s correlation coefficient was used to analyse the correlation between variables. *P* < 0.05 was considered statistically significant.

## Results

### Frequency of B Cells in Children with HSP

**Fig. 1:**
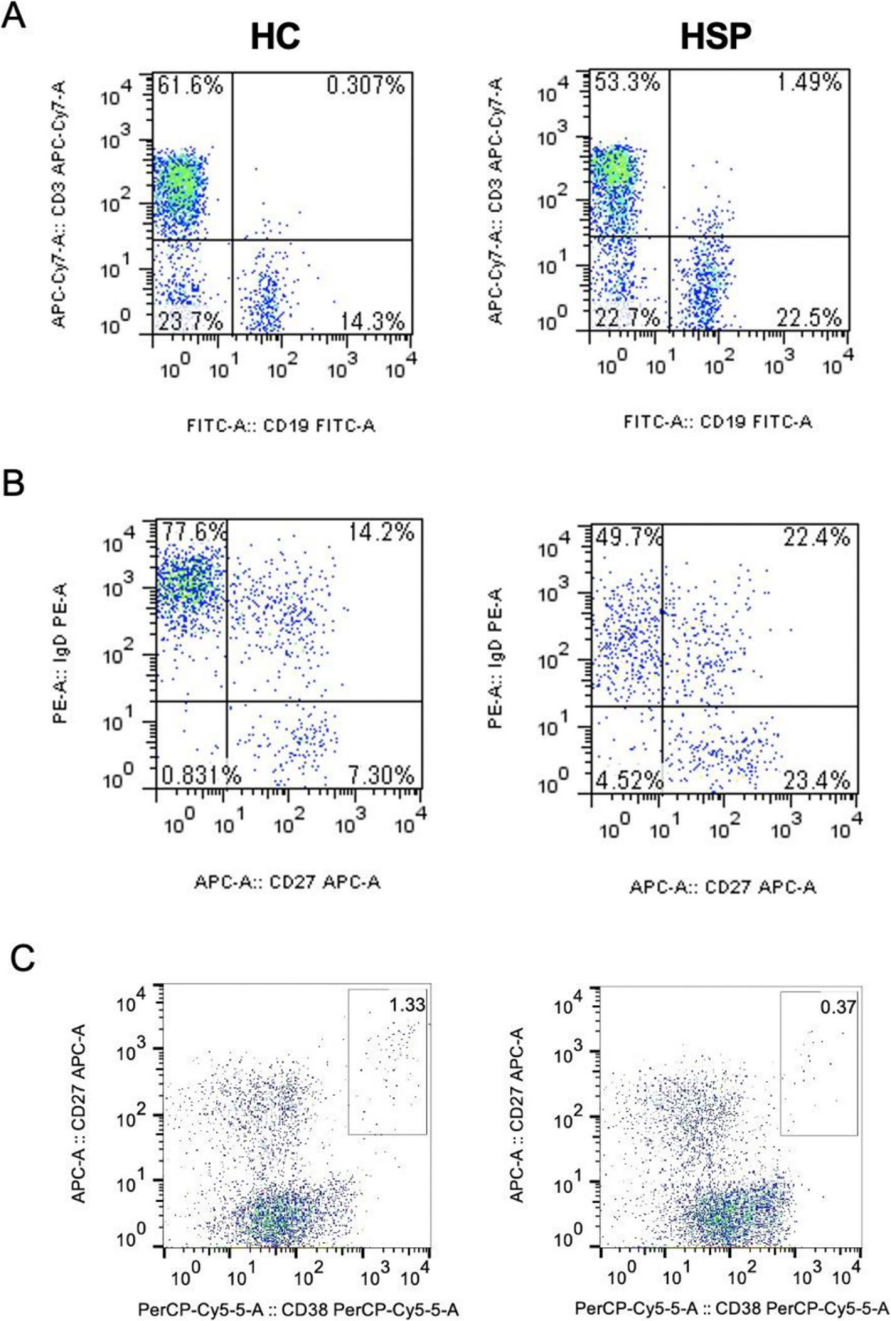
Flow cytometry analysis of the B cell compartment in children with HSP. The percentage of (**A**) CD3^−^CD19^+^ total B cells, (**B**) naïve B cells (IgD^+^CD27^−^), memory nonswitched B cells (IgD^+^CD27^+^) and memory switched B cells (IgD^−^CD27^+^), and (**C**) CD27^+^CD38^+^ plasma B cells in children with HSP compared with HCs. CD: cluster of differentiation, HC: healthy controls, HSP, Henoch-Schonlein purpura

**Fig. 2:**
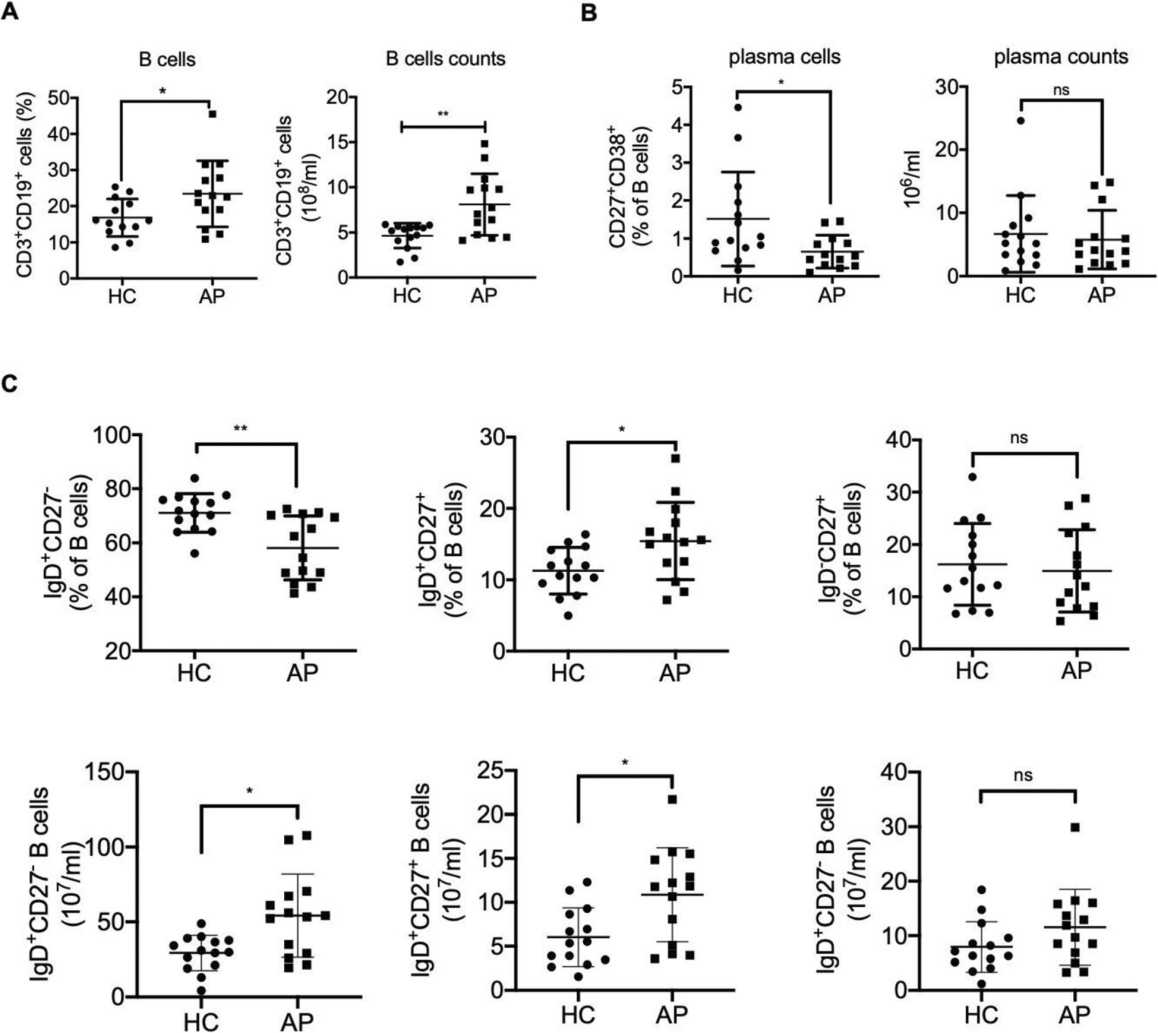
Analysis of the frequency and absolute number of B cell subsets in children with HSP. The percentage and absolute number of (**A**) CD3-CD19+ total B cells, (**B**) CD27++CD38++ plasma B cells and (**C**) naïve B cells (IgD+CD27-), memory nonswitched B cells (IgD+CD27+ ) and memory switched B cells (IgD-CD27+) in children with HSP compared with HC. Each dot represents an independent individual. * *p*<0.05, ** *p*<0.01, ns: not significantly different. CD: cluster of differentiation, HC: Healthy controls, AP: Acute Henoch-Schonlein Purpura.

To investigate the potential role of B cells in children with HSP, we isolated PBMCs from HSP patients and HCs. The total variability of the PBMCs was reported as > 95 %. We analysed the percentage and absolute counts of total CD3^−^CD19^+^ B cells, as well as of B-cell-related subsets, such as plasma cells (CD27^+^CD38^+^), naïve B cells (IgD^+^CD27^−^), memory nonswitched B cells (IgD^+^CD27^+^) and memory switched B cells (IgD^−^CD27^+^) (Fig. 1). As shown in Fig. 2 A, both the frequency and absolute counts of total B cells were significantly increased in children with HSP compared with the control group (frequency: *p* = 0.03; absolute counts: *p* = 0.004); however, the percentage, but not the absolute counts of plasma cells, were significantly lower in children with HSP (*p* = 0.03, Fig. 2B) than in the control group. Compared to the control group, the percentage of naïve B cells in children with HSP was significantly decreased, but the absolute number was significantly increased (frequency: *p* = 0.002; absolute counts: *p* = 0.01). Both the percentage and absolute number of memory nonswitched B cells were significantly increased in children with HSP (frequency: *p* = 0.02; absolute counts: *p *= 0.01) compared to the control group. There was no significant difference in the percentage and absolute number of memory-switched B cells between children with HSP and HCs (Fig. 2 C). Based on all the results, we speculated that altered B-cell subset proportions may occur in children with HSP.

### Increased Expression of CXCR5 on CD4^+^ T Cells in Children with HSP

**Fig. 3:**
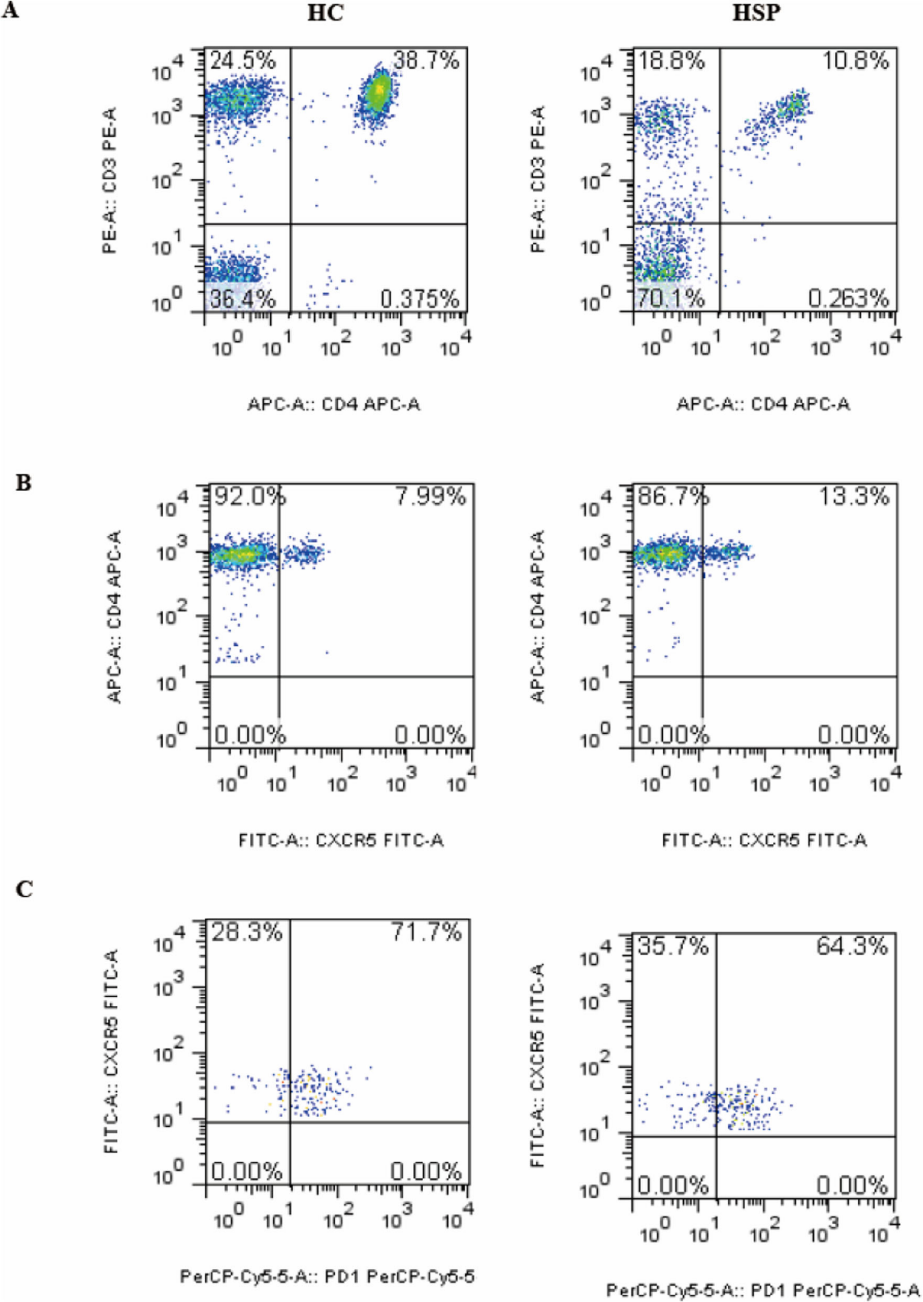
Analysis of the frequency of Tfh cells and PD-1 expression. Representative graph of the percentage of (**A**) The percentage of T cells, (**B**) CD4^+^CXCR5^+^ Tfh cells and (**C**) PD-1 expression on Tfh cells in children with HSP and HCs. CD: cluster of differentiation, HC: Healthy controls, HSP, Henoch-Schonlein purpura, Tfh, T follicular helper

**Fig. 4:**
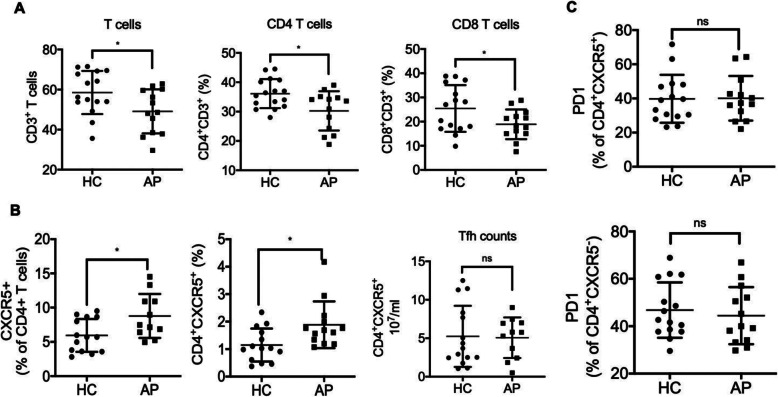
The frequency of Tfh cells and CXCR5 expression on total CD4^+^ T cells. (**A**) The percentage of peripheral CD3^+^, CD4^+^ and CD8^+^ T cells in children with HSP. (**B**) CXCR5 expression on total CD4^+^ T cells, the percentage and absolute number of Tfh cells in children with HSP. (**C**) PD-1 expression on CD4^+^CXCR5^+^ and CD4^+^CXCR5^−^ cells in children with HSP compared with HCs. Each dot represents an independent individual. ns: not significantly different, * *p* < 0.05. CD: cluster of differentiation, CXCR5, C-X-C chemokine receptor type 5, HC: Healthy controls, AP: Acute Henoch-Schonlein Purpura, PD-1, programmed cell death protein 1, Tfh, T follicular helper

Tfh cells, which provide specialized cognate help to B cells, were characterized as CXCR5- and PD-1-positive by flow cytometry[11]. Next, we used flow cytometry to determine whether the number of peripheral Tfh cells in children with HSP was increased compared to the control group (Fig. 3). We observed a decreased percentage of total CD3^+^, CD4^+^ and CD8^+^ T cells in children with HSP compared with the control group (Fig. 4 A). CXCR5 expression on total CD4^+^ T cells was significantly higher in HSP patients (*p* = 0.02, Fig. 4B) than in the control group. Compared to the control group, the percentage of CD4^+^CXCR5^+^ cells among total lymphocytes was significantly increased in HSP patients (*p* = 0.01), but the absolute number of Tfh cells was not different (Fig. 4B). Moreover, neither PD-1 expression on CD4^+^CXCR5^+^ cells nor the percentage of CD4^+^CXCR5^−^ cells in total lymphocytes differed significantly between patients with HSP and HCs (Fig. 4 C).

### Correlation of B-Cell Subsets with Tfh Cells in Children with HSP

**Fig. 5:**
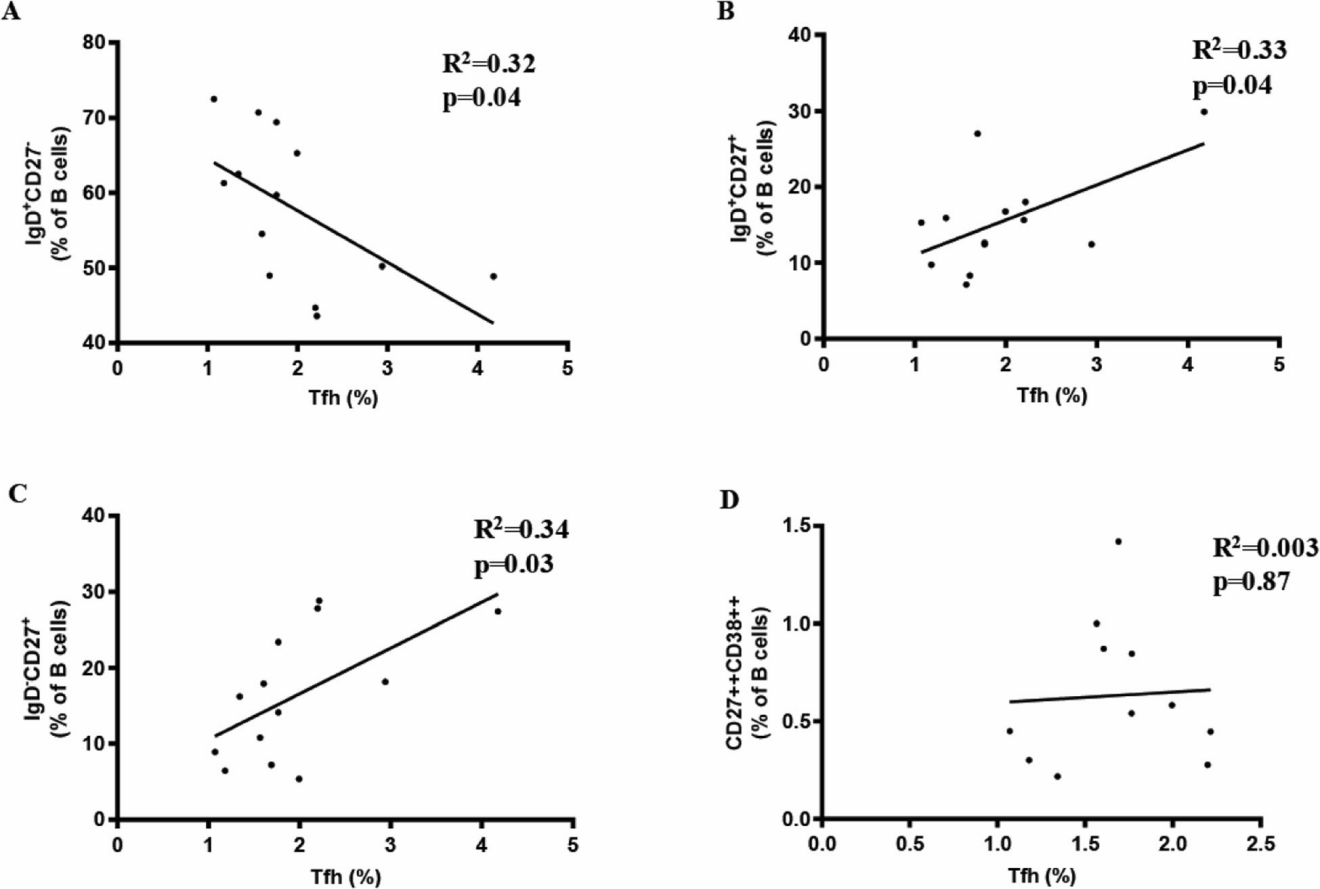
Correlation between of B cells subsets and Tfh cells. Correlation analysis of (**A**) IgD^+^CD27^−^ B cells, (**B**) IgD^+^CD27^+^ B cells, (**C**) IgD^−^CD27^+^ B cells and (**D**) CD27^++^CD38^++^ plasma B cells with the percentage of CD4^+^CXCR5^+^ Tfh cells or CXCR5 expression on total CD4^+^ T cells. CD: cluster of differentiation, CXCR5, C-X-C chemokine receptor type 5, Tfh, T follicular helper

We measured the B-cell proportions (as percentages) in the 14 patients included in the study. However, unforeseen events prevented us from obtaining Tfh-cell data for one patient. Therefore, the sample size for the data presented in Fig. 5 A-C is 13. As the percentage of CD27^+^CD38^+^ plasma cells from two other patients was nearly 0, the data for these two patients has been excluded from Fig. 5D. Correlation analysis revealed that the percentage of CD4^+^CXCR5^+^ cells was significantly correlated with the percentage of total CD3^−^CD19^+^ B cells (Fig. 3 A). Furthermore, CXCR5 expression on total CD4^+^ T cells and the percentage of CD4^+^CXCR5^+^ cells was significantly correlated with the percentage of naïve B cells (IgD^+^CD27^−^), memory nonswitched B cells (IgD^+^CD27^+^) and memory-switched B cells (IgD^−^CD27^+^); however, no corresponding correlation with plasma cells was observed (Fig. 5). We divided the HSP group into two based on whether patients exhibited complications from renal impairment but found no differences in the percentage and absolute numbers of B or Tfh cells between the two groups (data not shown).

## Discussion

Emerging evidence has shown that HSP is a systemic disease in children; however, the pathogenesis of HSP remains unknown. Functional mutations of T cells, such as abnormal cytokine secretion, have been reported to be involved in the pathogenesis of HSP[12]. Increased IgA indicates that humoral immunity underlies the manifestations of HSP; however, whether altered proportions of B-cell subsets and Tfh-dependent B cell responses play a role in the pathogenesis of HSP remains unknown.

HSP, also characterized as IgA vasculitis, is the most common vasculitis in children[13]. Recently, Courtney et al. retrospectively analysed eight children treated with RTX (rituximab, a B cell-depleting agent) and found that RTX treatment effectively helped most children achieve clinical remission[14]. However, to the best of our knowledge, no studies have been conducted to investigate the populations of total B cells and plasma cells in association with HSP. In the present study, we found that the percentage and absolute number of total B cells were significantly increased in children with HSP compared with HCs. However, the percentage of CD27^+^CD38^+^ plasma cells was significantly reduced in children with HSP compared with HCs. This study is the first report of increased total B cells and a reduced frequency of plasma cells in children with HSP. Further study is needed to delineate the detailed mechanisms of how increased total B cells and the reduced frequency of plasma cells contribute to IgA vasculitis in HSP children.

Functionally distinct B-cell subsets can be divided into different subsets by the phenotypic expression of CD27 and IgD. IgD^+^CD27^−^ B cells have been defined as naïve B cells, whereas CD27 expression by B cells has been considered a hallmark of memory. CD27^+^ memory B cells can also be divided into memory nonswitched (IgD^+^CD27^+^) and memory switched (IgD^−^CD27^+^) B cell subsets. We sought to determine whether the B-cell compartment of children with HSP differed from that of controls, and our results showed that in children with HSP, the percentage of naïve B cells was significantly reduced, whereas that of memory nonswitched B cells was significantly increased. Our results indicated that altered B-cell subsets might be related to IgA vasculitis in children with HSP. Previous studies demonstrated that a reduction in interleukin-10 (IL-10)-producing regulatory B cells (B10 cells) contributes to the pathogenesis of HSP nephritis[5, 15]. However, in the present study, we divided the HSP group into two based on whether patients exhibited complications from renal impairment and found no differences in the percentage and absolute numbers of total B cell and B-cell subsets between the two groups.

A large number of studies have shown that Ig production is associated with the frequency of Tfh cells in a variety of autoimmune diseases, such as rheumatoid arthritis and systemic lupus erythematosus[16, 17]. We found significant increases in the expression of CXCR5 on CD4^+^ T cells and the total frequency of CXCR5^+^CD4^+^ Tfh cells in children with HSP compared with HCs. There was no difference in the expression of PD-1, a functional marker of Tfh cells, between the two groups. Similar results have been previously reported, that is, the expansion of circulating Tfh cells in children with acute HSP, a significant increase in CXCR5^+^CD4^+^ cells in children with HSP and no differences in PD-1 expression between children with HSP and controls[18]. Moreover, Tfh cells were found to be correlated with naïve B cells, memory nonswitched B-cell subsets, and memory switched B cells in children with HSP in our study. Our findings imply that an increased percentage of Tfh cells might contribute to abnormal B cell class switching in the germinal centre, which eventually results in increased IgA production in children with HSP.

This study may have some limitations. The small sample used in this study may have introduced a statistical bias. Large-scale studies on children with HSP are required to address this potential limitation. Moreover, lymphoid tissues could not be obtained from children with HSP, and we only determined the frequencies of circulating B cells and Tfh cells in the peripheral blood of children with HSP.

In summary, our study showed altered circulating B-cell subsets and a Tfh cell-related altered B-cell compartment in children with HSP. Our findings may provide new insight into the pathogenesis of children with HSP.

**Table 1:** Demographic and Basic Laboratory Characteristics of HSP patients and HCs

	HSP	HCs
Number	14	14
Age (years)	7.5 ± 2.1	7.2 ± 1.9
Sex (M/F)	8/6	7/7
WBC (⋅ 10^9^)	11.5 ± 2.3	6.5 ± 1.8
Lymphocytes (⋅ 10^9^)	3.52 ± 0.89	2.72 ± 0.85
Serum IgA (g/dL)	1.98 ± 0.92	1.77 ± 0.53
Serum IgG (g/dL)	10.56 ± 3.42	9.34 ± 2.52
Serum IgM (g/dL)	1.45 ± 0.51	1.13 ± 0.47

## Data Availability

The datasets used and analysed during the current study available from the corresponding author on reasonable request.

## References

[1] Basaran O, et al. Plasma exchange therapy for severe gastrointestinal involvement of Henoch Schonlein purpura in children. Clin Exp Rheumatol. 2015;33:–176. 25436762

[2] Chen JY, Mao JH (2015). Henoch-Schonlein purpura nephritis in children: incidence, pathogenesis and management. World journal of pediatrics: WJP.

[3] Jen HY, Chuang YH, Lin SC, Chiang BL, Yang YH (2011). Increased serum interleukin-17 and peripheral Th17 cells in children with acute Henoch-Schonlein purpura. Pediatric allergy immunology: official publication of the European Society of Pediatric Allergy Immunology.

[4] Yi H (2007). [Effect of Shenyankangfu tablet on urinary IL-6 and its therapeutic effect in children with Henoch-Schonlein purpura nephritis]. Zhongguo dang dai er ke za zhi = Chinese journal of contemporary pediatrics.

[5] Yang B (2017). Effect of CD40/CD40L signaling on IL-10-producing regulatory B cells in Chinese children with Henoch-Schönlein purpura nephritis. Immunol Res.

[6] Gonzalez-Gay MA, Blanco R, Castaneda S (2017). Henoch-Schonlein purpura (IgA vasculitis): the paradox of the different incidence and clinical spectrum in children and adults. Clin Exp Rheumatol.

[7] Lin W (2014). B cell subsets and dysfunction of regulatory B cells in IgG4-related diseases and primary Sjogren’s syndrome: the similarities and differences. Arthritis research therapy.

[8] Nakayamada S, Tanaka Y (2016). T follicular helper (Tfh) cells in autoimmune diseases. Nihon Rinsho Men’eki Gakkai kaishi = Japanese journal of clinical immunology.

[9] Jeon YH, Choi YS, Follicular Helper T (Tfh) Cells in Autoimmune Diseases and Allograft Rejection. *Immune network* **16**, 219–232 (2016). 10.4110/in.2016.16.4.219 PMC5002448 27574501

[10] Yang YH, Yu HH, Chiang BL (2014). The diagnosis and classification of Henoch-Schonlein purpura: an updated review. Autoimmun rev.

[11] Kim AR (2020). Targeting inducible costimulator expressed on CXCR5(+)PD-1(+) T(H) cells suppresses the progression of pemphigus vulgaris. J Allergy Clin Immunol.

[12] Huang DL (2011). [Relationship between renal Th1/Th2 ratio and renal microvascular injury in children with Henoch-Sch-nlein purpura nephritis]. Zhongguo dang dai er ke za zhi = Chinese journal of contemporary pediatrics.

[13] Roman C, Dima B, Muyshont L, Schurmans T, Gilliaux O (2019). Indications and efficiency of dapsone in IgA vasculitis (Henoch-Schonlein purpura): case series and a review of the literature. Eur J Pediatrics.

[14] Crayne CB (2018). Rituximab treatment for chronic steroid-dependent Henoch-Schonlein purpura: 8 cases and a review of the literature. Pediatr Rheumatol Online J.

[15] Hu X (2016). A Lower Proportion of Regulatory B Cells in Patients with Henoch-Schoenlein Purpura Nephritis. PLoS One.

[16] Cao G, Chi S, Wang X, Sun J, Zhang Y (2019). CD4 + CXCR5 + PD-1 + T Follicular Helper Cells Play a Pivotal Role in the Development of Rheumatoid Arthritis. Medical science monitor: international medical journal of experimental clinical research.

[17] Bocharnikov AV, et al. PD-1hiCXCR5- T peripheral helper cells promote B cell responses in lupus via MAF and IL-21. *JCI insight* 4 (2019). 10.1172/jci.insight.130062 PMC6824311 31536480

[18] Xie J (2015). Expansion of Circulating T Follicular Helper Cells in Children with Acute Henoch-Schönlein Purpura. Journal of Immunology Research.

